# Can Foot Dermatophyte Infections Signal Future Diabetes Risk? Findings from a Register-based Study

**DOI:** 10.2340/actadv.v106.adv-2025-0125

**Published:** 2026-03-09

**Authors:** Anne Sofie Frølunde, Jan Brink Valentin, Lise Kristensen, Pernille Kræmer Schachsen, Janus Laust Thomsen, Christian Vestergaard

**Affiliations:** 1 Department of Dermatology, Aarhus University Hospital, Aarhus, Denmark; 2 Aarhus University, Aarhus, Denmark; 3 Center for General Practice, Aalborg University, Aalborg, Denmark; 4 Department of Clinical Microbiology, Aarhus University Hospital, Aarhus, Denmark

**Keywords:** Onychomycoses 1, Tinea pedis 2, Diabetes Mellitus, Type 2 3

## Abstract

Dermatophyte infections are common in general practice and occur more often in individuals with type 2 diabetes (T2D), but whether they signal undiagnosed T2D remains unclear. We conducted a register-based cohort study including positive PCR tests for dermatophyte infection from the feet or nails, matched 1:3 to individuals from the same geographic area in Denmark. Those with known diabetes, type 1 diabetes or aged under 20 were excluded. Incidence rates (IRs) and incidence rate ratios (IRRs) for new-onset T2D were estimated using Poisson regression. The final cohort comprised 78,140 individuals, with a median age of 51 years, and 60.8% were male. The IR for T2D was 9.23 per 100 person-years in the exposed group and 9.00 in the unexposed group, with an adjusted IRR of 1.00 (0.91–1.11, *p*=0.94), indicating no significant association. In a sensitivity analysis excluding unexposed individuals with prior topical antifungal treatment, the IRR increased to 1.15 (1.08–1.23, *p*=0.001). While the primary analysis showed no significant association, the sensitivity analysis suggested a modest increased risk when exposure misclassification was reduced, supporting dermatophyte infection as a possible early signal of undiagnosed T2D in selected populations.

**SIGNIFICANCE**

Fungal infections of the feet and nails are common in general practice and often occur in people with type 2 diabetes. We asked whether such infections could be an early warning sign of undiagnosed diabetes. In a large Danish cohort, PCR-confirmed infections were not clearly linked to later diabetes overall. However, when we reduced likely misclassification (by excluding unexposed people who had used antifungal creams), the risk was modestly higher. These findings suggest that targeted diabetes assessment may be relevant in selected patients, rather than routine screening of everyone with a fungal infection.

Skin manifestations can serve as early, clinically accessible indicators of systemic disease, including type 2 diabetes (T2D) (1–3). Dermatological signs may precede the diagnosis of T2D by years and provide a valuable, low-cost opportunity to detect underlying metabolic dysfunction at an early stage.

T2D is a chronic metabolic disorder characterized by insulin resistance, impaired insulin secretion and persistent hyperglycaemia (4). According to the International Diabetes Foundation, an estimated 536.6 million people were living with diabetes, diagnosed or undiagnosed, in 2021, a number projected to rise by 46% to 783.2 million by 2045 (5). T2D frequently remains undiagnosed for several years, often until complications have developed (6–8). It is estimated that up to 50% of individuals with T2D are unaware of their condition and that metabolic abnormalities may be detectable long before formal diagnosis (7–9).

The metabolic disturbances characteristic of T2D are associated with a range of cutaneous changes. Chronic hyperglycaemia has been shown to affect the skin’s microenvironment by increasing surface pH, impairing immune responses and disrupting keratinocyte function and epidermal barrier integrity (2, 3, 10). These changes are believed to increase susceptibility to infections, including superficial fungal infections such as dermatophytosis (11–13).

Dermatophytes constitute a group of keratinophilic fungi that are capable of colonizing and invading the outermost keratinized tissues of the human body, including the skin, hair and nails (14, 15). They are highly contagious and among the most common fungal pathogens globally (14, 16). *Trichophyton rubrum* is the most frequently identified dermatophyte species and remains the most common causative agent of dermatophytosis globally (17). The global prevalence of toenail onychomycosis is estimated at 4%, with higher rates in specific populations (18), while tinea pedis affects up to 70% of individuals at some point in life (19). Dermatophyte infections are classified clinically according to anatomical site (tinea corporis, tinea pedis or tinea unguium) and have been found to be more prevalent in people with diabetes (20–22), likely due to a combination of the impaired immune function, vascular complications and a skin microenvironment that favours fungal proliferation.

A 2017 Finnish study by Sinikumpu et al. explored whether toe web changes, such as maceration, vesicles and redness, were associated with abnormal glucose metabolism in adults aged 45–46 years (23). In this population-based cohort of 1,849 individuals, such subtle dermatological changes were significantly associated with metabolic abnormalities, suggesting a potential role for skin signs as early markers of T2D risk (23) and thus holding potential as screening tools in primary care.

Inspired by these findings, the present study investigates whether dermatophyte infections of the feet and nails, confirmed by routine PCR testing, may be associated with either undiagnosed T2D at the time of testing or an increased risk of receiving a T2D diagnosis in the following years. Given that both dermatophyte infections and T2D are commonly encountered in general practice, microbiologically confirmed dermatophyte infections could represent a useful, low-cost and widely accessible tool for early identification of patients at risk of diabetes.

## MATERIALS AND METHODS

### Study design and setting

This registry-based, matched cohort study used data from the Department of Clinical Microbiology’s Data System (MADS), a Danish laboratory information system for managing and reporting clinical microbiology results in the Central Denmark Region, linked with data from nationwide health registries from the Danish Health Data Authority. The study population comprised residents of the Central Denmark Region, as MADS data access was limited to this area.

Denmark operates a universal, tax-funded healthcare system that provides comprehensive access to both primary and secondary healthcare services. Each citizen is assigned a unique civil registration number, enabling individual-level linkage across multiple healthcare registers (24).

The study was approved by the Danish Data Protection Agency, Central Denmark Region (approval no. 1-45-70-86-22).

### Study population

We included individuals aged ≥20 with a positive PCR test for dermatophyte infection affecting the feet, toe web spaces or toenails, as recorded in MADS (exposed group). Each exposed individual was matched 1:3 on birth year and sex to unexposed controls drawn from the general population of the Central Denmark Region. The date of the PCR test was used as the index date assigned to matched controls.

Individuals were excluded if they were under 20 years of age, due to the higher prevalence of dermatophyte infections from environmental exposures in this group (e.g. such as sports activities and shared public facilities). To ensure data completeness and reliability, individuals were required to have resided in Denmark for at least 1 year prior to the index date.

### Data sources

Data for this study were obtained from 4 national health registries and the MADS database. The local MADS at the Department of Clinical Microbiology at Aarhus University Hospital provided PCR test results for dermatophyte infections in the Central Denmark Region from March 29, 2016, through 2021. PCR for dermatophytes was performed using an in-house real-time multiplex assay including pan-dermatophyte detection and specific targets for the 2 most common dermatophytes causing skin and nail infections, *T. rubrum* and *Trichophyton interdigitale/mentagrophytes*. Pan-dermatophyte positive samples without species identification were further analysed using a commercial kit (DermaGenius^®^ 2.0, PathoNostics) covering a broader range of dermatophyte species (*Trichophyton violaceum, Epidermophyton floccosum, Trichophyton benhamiae, T. interdigitale/T. mentagrophytes, T. rubrum/soudanense, Trichophyton tonsurans, Trichophyton verrucosum, Microsporum canis and Microsporum audouinii*).

Prescription data were retrieved from the Danish National Prescription Registry, which has recorded all pharmacy-dispensed prescriptions in Denmark since 1994 (24, 25). Information on hospital admissions, emergency room visits and outpatient contacts was obtained from the Danish National Patient Registry (DNPR), which has recorded diagnoses using ICD-10 codes since 1994 (24, 26). Laboratory test results, including HbA1c measurements, were obtained from the Clinical Laboratory Information System (LABKA), a comprehensive repository of test results from both primary and secondary healthcare sectors (27). Finally, demographic information, including age, sex, residency status and vital status, was collected from the Danish Civil Registration System, which allows for accurate individual-level linkage across registries (28).

### Outcome

Diabetes was classified based on hospital diagnoses and prescription records as the primary outcome, while an additional classification incorporating HbA1c measurements was used as a secondary outcome for sensitivity analysis.

For the primary outcome, participants were classified as having diabetes if they had either a recorded diagnosis in the DNPR with an ICD-10 code for diabetes (E10-E15) or a diabetes-related complication (DO24, DT383A, DM142, DG590, DG632, DH280, DH334, DH450, DH360, DN083), whichever occurred first, or if they had a prescription for glucose-lowering medication (ATC code starting with A10). If both prescription data and hospital diagnoses were available, the earliest recorded event was used as the diagnosis date (29).

To assess the robustness of our findings, we also conducted a sensitivity analysis using an expanded diabetes definition. For this secondary outcome, participants were classified as having diabetes if they met the primary outcome criteria or had at least 2 HbA1c measurements above 48 mmol/mol within 1 year. In an additional sensitivity analysis, individuals in the unexposed group who had received topical antifungal treatment prior to the index date were excluded. To preserve the matched design, corresponding cases were also removed if none of their matched controls remained.

Type 1 diabetes was defined as ICD-10 code E10, while T2D was classified under E11. Individuals with any diabetes diagnosis prior to the index date were excluded, along with their matched controls.

### Exposure

The primary exposure was one or more positive dermatophyte PCR test results from toenails, toe web spaces or feet. The unexposed group consisted of matched individuals without such infections.

### Comorbidities

Comorbidities were assessed using the Charlson Comorbidity Index (CCI), based on ICD-10 diagnoses in DNRP. To ensure a comprehensive assessment of comorbidity burden, diagnoses were included from 5 years before to 5 years after the index date. Diabetes was excluded from the CCI calculation to avoid overlap with the primary outcome of the study. Each participant was assigned a CCI score, which was categorized into 4 standard risk groups for statistical analysis: 0, 1–2, 3–4 and ≥5.

### Statistical analyses

**Fig. 1.**
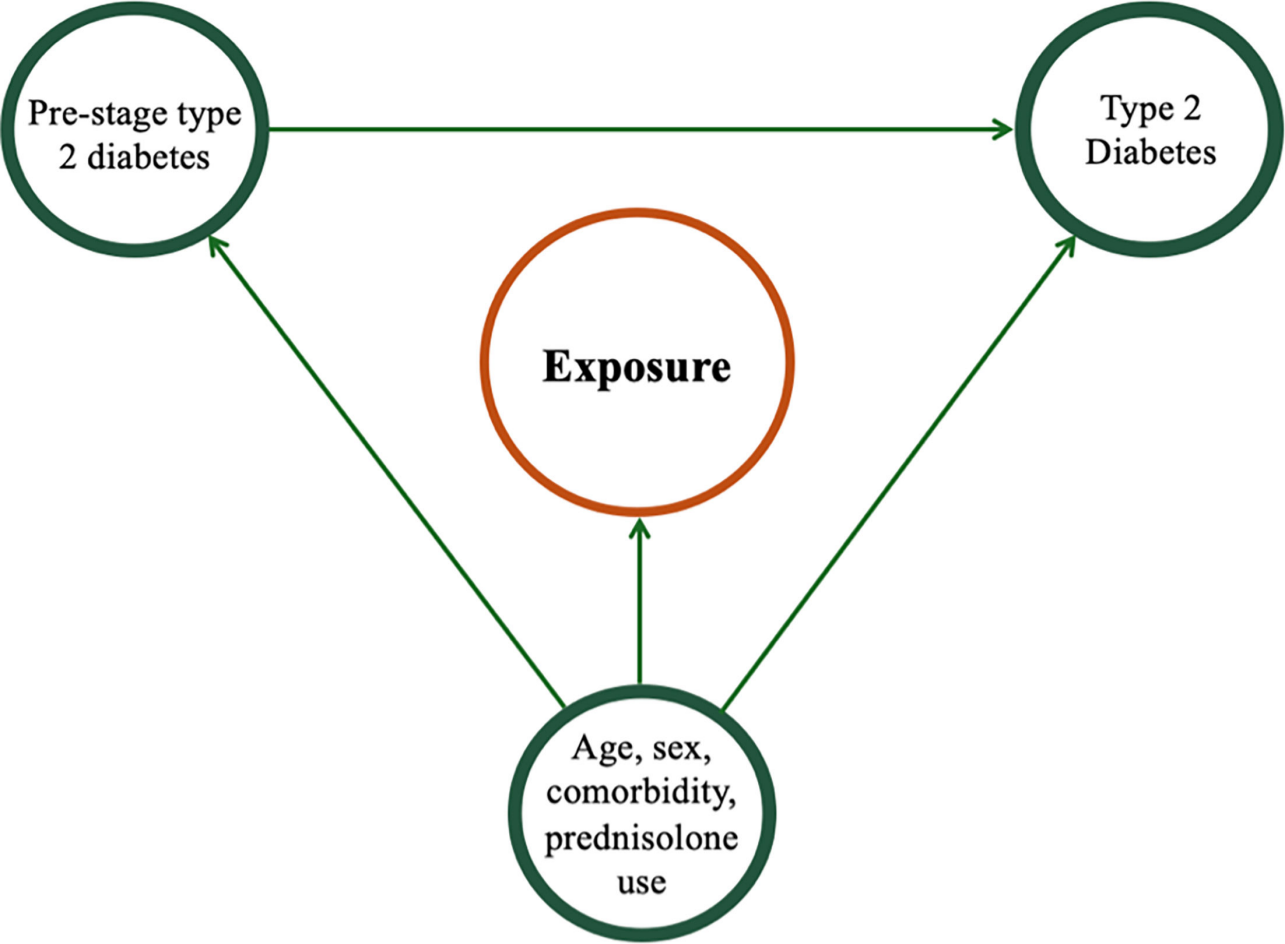
Causal diagram illustrating hypothesized relationship between exposure, confounding factors (age, sex, comorbidities and prednisolone use), pre-stage diabetes and the development of type 2 diabetes.

Descriptive statistics were used to summarize demographic and clinical characteristics of all individuals. For the primary exposure, we employed Poisson regression analyses to estimate the incidence rate ratio (IRR) from the index. Cluster-robust variance estimation was applied with each match group constituting a cluster. To investigate the influence of age and sex, subgroup analyses were conducted stratified by age and sex groups. Because we expect dermatophyte infection to be a predictor of diabetes without having a causal relation, we adjusted for age, sex, prednisolone use and comorbidity, but not pre-stage diabetes (see Fig. 1). The effect of the secondary exposure on diabetes will be investigated similarly, but without applying cluster-robust variance estimation. Results are presented with 95% confidence intervals (CI). All data management and statistical analyses were performed in StataSE version 18.

## RESULTS

### Baseline characteristics of the study (Table I).

**Table I.** Baseline characteristics of the study population

Variable	Exposed (n=18,785)	Unexposed (n=59,355)
Demographic characteristics		
Age, years (median, IQR)	51 (39–63)	51 (39–63)
Age group (*n*, (%))		
20–39 years 40–59 years 60–79 years 80 years and above	4,830 (25.7) 7,991 (42.5) 5,241 (27.9) 723 (3.8)	14,492 (25.7) 24,020 (42.5) 15,639 (27.8) 2,204 (3.9)
Gender (*n*, (%))		
Male Female	11,412 (60.8) 7,373 (39.2)	34,234 (60.7) 22,119 (39.3)
Clinical characteristics		
Prevalence of T2D, *n* (%)	1,752 (9.4)	5,049 (9.0)
Comorbidities (*n*, (%))		
Cardiovascular disease (heart failure) Hypertension Moderate or severe chronic kidney disease Any malignancy	224 (4.8) 610 (13.1) 271 (5.8) 1,088 (5.5)	840 (6.4) 1,752 (13.3) 768 (5.8) 3,304 (5.6)
Charlson Comorbidity Index	1 (1–2)	1 (1–2)
Diagnosing physician		
General practitioner Practising specialist Medical doctor at hospital department	88.8% 8.7% 2.6%	N/A
Localization of infection		
Skin scraping from feet or toes Nail sample	28.5% 71.6%	N/A
Identified dermatophyte species		
*Trichophyton rubrum* *Trichophyton interdigitale* *Trichophyton violaceum* *Epidermophyton floccosum*	80.2% 17.5% 0.00016% 0.00077%	N/A

IQR:Interquartile Range; n:number; T2D:Type 2 Diabetes.

The final cohort comprised 78,140 individuals, including 18,785 in the exposed group and 59,355 in the unexposed group. Median age was 51 years (interquartile range (IQR): 39–63) in both groups, and 60.8% were male. The prevalence of comorbidities, such as hypertension, heart failure, kidney disease and malignancy, was low overall and comparable between the groups. Similarly, the distribution of CCI scores showed no substantial differences between exposed and unexposed individuals. Most PCR tests were ordered in general practice (88.8%), nail samples were the most predominant specimen type (71.6%), and *T. rubrum* was the most frequently identified dermatophyte species (80.2%).

### The effect of a positive dermatophyte PCR test on the development of type 2 diabetes (Table II).

The incidence of newly diagnosed T2D was 9.23 (95% CI: 8.80–9.66) per 100 person-years in the exposed group and 9.0 (95% CI: 8.75–9.25) per 100 person-years in the unexposed group, corresponding to an unadjusted IRR of 1.03 (0.97–1.08). After adjustment for age, sex, comorbidities and systemic prednisolone use, the IRR was 1.00 (95% CI: 0.91–1.11), indicating no statistically significant association between a positive dermatophyte PCR test and subsequent development of T2D.

Additional analyses showed no dose–response association according to number of positive dermatophyte PCR tests and no significant association with development of prediabetes (Tables SI–SII)

**Table II.** Risk of type 2 diabetes following a positive PCR test for dermatophyte infection

Variable	Primary outcome per 100 person-years with 95% CI	IRR with 95% CI	*p*-value
IR (unexposed)	9.00 (8.75–9.25)	Ref.	
IR (exposed)	9.23 (8.80–9.66)	1.03 (0.97–1.08)	0.35
Adjusted model*	-	1.00 (0.91–1.11)	0.94

IRs are based on predictive margins from unadjusted Poisson regression models with time-to-event as the exposure variable.

*Adjusted for age, sex, hypertension, kidney disease, heart failure, malignancy and systemic prednisolone use.

CI:Confidence Interval; IR:Incidence rates.

### Sensitivity analysis (Table III)

In the first sensitivity analysis, the diabetes definition was expanded to include individuals with at least 2 HbA1c measurements ≥48 mmol/mol within 1 year. This broader definition led to slightly higher incidence rates (IRs) in both exposed and unexposed groups, but the IRR remained close to unity (IRR=1.05, 95% CI: 0.99–1.11, *p*=0.08).

**Table III.** Sensitivity analyses of type 2 diabetes definitions

Variable	Primary outcome^a^ per 100 person-years with 95% Confidence Interval (CI)	Secondary outcome^b^ per 100 person-years with 95% Confidence Interval (CI)	Secondary outcome^c^ per 100 person-years with 95% Confidence Interval (CI)
IR unexposed group	9.00 (8.75–9.25)	9.1 (8.9–9.40)	7.98 (7.67–8.30)
IR exposed group	9.23 (8.80–9.66)	9.6 (9.1–10.0)	9.12 (8.74–9.64)
IRR	1.03 (0.97–1.08)	1.05 (0.99–1.11)	1.15 (1.08–1.23)
*p*-value	0.35	0.08	0.001

^a^Diabetes defined with a hospital diagnosis OR antidiabetic medication*.* ^b^Diabetes defined with a hospital diagnosis OR antidiabetic medication OR 2 HbA1c measurements ≥48 mmol/mol in 1 year. ^c^Diabetes defined with a hospital diagnosis OR antidiabetic medication, and where patients with prior topical antimycotic treatment has been excluded from the unexposed group.

In a second sensitivity analysis, individuals in the control group who had received topical antifungal treatment prior to the index date were excluded. Under this restriction, a statistically significant association emerged between a positive dermatophyte PCR test and subsequent T2D (IRR=1.15, 95% CI: 1.08–1.23, *p*=0.001), with IR of 9.12 and 7.98 per 100 person-years in the exposed and unexposed groups, respectively. These findings suggest that misclassification of exposure in the control group may have attenuated the association in the primary analysis.

## DISCUSSION

The central finding of our study showed no statistically significant association between PCR-confirmed dermatophyte infection of the feet or nails and subsequent risk of developing T2D in the primary analysis. This contrasts with the findings by Sinikumpu et al. (21), who reported that toe web abnormalities, such as maceration, erythema and vesicles, were associated with screen-detected diabetes and elevated HbA1c levels. The authors speculated that tinea pedis could explain many of these skin changes, although no microbiological testing was performed.

A key difference between the 2 studies lies in the exposure definition. Sinikumpu et al. prioritized identifying visible skin changes, arguing that these may be more relevant for preventive efforts than laboratory-confirmed diagnoses. In contrast, we defined our exposure as a positive PCR test for dermatophyte infection, as fungal infections are the most common skin infections among patients with T2D. This choice was further supported by the clinical descriptions provided in the Finnish study, where toe web changes were characterized as maceration, scaling, vesicles and localized erythema, features that are commonly seen in tinea pedis caused by dermatophytes (19). We also included onychomycosis in our exposure definition, as approximately one-third of patients with tinea pedis also have nail involvement. The majority of these infections were caused by *T. rubrum* or *T. interdigitale (19, 30*). However, it is important to note that other microorganisms may also contribute to skin changes in the toe web spaces of diabetic patients. A clinical study from Turkey comparing patients with T2D with healthy controls found *Trichophyton* species to be the most common pathogens but also identified non-dermatophyte fungi such as *Trichosporon*, *Fusarium* and *Aspergillus* spp (12). Bacterial infections can likewise be potential causes of skin changes. Importantly, this study reported no significant association between dermatophyte infection and HbA1c levels, duration of diabetes or body mass index (BMI). In contrast, a recent study from India identified a positive correlation between HbA1c levels and the number of affected nails in patients with onychomycosis (22).

Obesity is a shared risk factor for both fungal infections and T2D (31, 32). Due to data limitations in Danish health registries, we were unable to adjust for BMI. The Finnish study collected height and weight data, enabling calculation of BMI. They observed a significantly higher proportion of overweight and obese individuals among those with toe web changes and adjusted for this in their statistical models. Even after adjustment, the associations between skin changes and both oral glucose tolerance test (OGTT)-defined diabetes and elevated HbA1c remained statistically significant (23).

Low socioeconomic status is also a known risk factor for both obesity and T2D, particularly among women in high-income countries (32). This may have implications for our study, as it includes only individuals who actively sought medical care for foot or nail symptoms. Consequently, our findings reflect a subset of patients who were both able to recognize dermatological symptoms and motivated to consult a healthcare provider, whereas the Finnish study relied on a pre-defined population invited for clinical examination, thereby minimizing selection bias related to healthcare-seeking behaviour and potentially capturing a broader cross-section of the population. These differences in study design and population selection may help explain the divergent findings, as our study may under-represent individuals with lower health literacy, groups potentially at higher risk of undiagnosed T2D.

The 2 studies differ substantially in design and methodology. Our primary strength lies in the large, register-based cohort of nearly 18,785 individuals with laboratory-confirmed dermatophyte infection. In contrast, the Finnish study included only 492 participants assessed via clinical examination, with small subgroups yielding statistically significant associations (23). The screening-detected diabetes group with toe web changes included 24 patients, while the elevated HbA1c group consisted of just 9 cases. Thus, while significant, these results should be interpreted cautiously due to limited sample size.

Importantly, our sensitivity analyses revealed a more nuanced picture. When using an expanded definition of diabetes that included elevated HbA1c, a trend toward increased risk emerged. Furthermore, when excluding individuals in the unexposed group who had received prior topical antifungal treatment, a statistically significant association was observed (IRR 1.15; 95% CI: 1.08–1.23). This stepwise increase in risk across definitions, from 1.01 to 1.05 to 1.15, suggests that exposure misclassification may have attenuated a true underlying association in the main analysis. These findings imply that dermatophyte infections may hold clinical relevance as potential indicators of metabolic dysfunction in selected patient groups.

### Limitations

This study has several limitations. First, although we relied on high-quality national health registers, some degree of misclassification of both exposure and outcome cannot be excluded. Dermatophyte infections are often treated empirically in primary care without microbiological testing, and such cases would not appear in our dataset. Similarly, individuals with undiagnosed or untreated infections may have been misclassified as unexposed.

Second, although we adjusted for a range of comorbidities, residual confounding may remain. In particular, obesity is a major shared risk factor for both dermatophyte infections and T2D. As BMI is not routinely recorded in Danish registers, we were unable to adjust for this variable.

Third, individuals in the unexposed group may have had unrecognized toe web abnormalities or onychomycosis that were never tested, potentially diluting the observed association. This is supported by our sensitivity analysis, which demonstrated a significant association once individuals with prior antifungal treatment were excluded from the control group.

Furthermore, atopy represents a potential residual confounder not accounted for in our analysis. Atopic dermatitis (AD) has been associated with both an increased susceptibility to tinea pedis and a higher risk of developing T2D (33, 34). Recent evidence suggests a significant correlation between AD and tinea pedis, while separate meta-analyses have linked AD to metabolic disturbances, including T2D. While we adjusted for a range of comorbidities, the lack of specific data on atopic status in the registries means we cannot rule out its role as a predisposing factor for both the fungal exposure and the metabolic outcome.

Finally, although HbA1c data were incorporated into a sensitivity analysis, some individuals with undiagnosed diabetes may not have undergone testing and thus were not captured in the dataset.

### Conclusions

In this large, register-based cohort study, we found no evidence of an increased risk of developing T2D among individuals with PCR-confirmed dermatophyte infections of the feet, including toenail infections, compared with unexposed controls. The null findings were consistent across subgroup analyses and when applying an extended diabetes definition based on HbA1c values.

However, in a refined sensitivity analysis excluding controls with prior antifungal treatment, a statistically significant association emerged, suggesting that exposure misclassification may have attenuated the observed association in the primary analysis.

Although our results contrast with those from Sinikumpu et al. (23), differences in study design, exposure definition and population characteristics may explain the discrepancy. In conclusion, while our primary analysis showed no significant association, the sensitivity analyses suggest that dermatophyte infections may signal metabolic dysfunction in certain patient subgroups. However, it is premature to incorporate these findings into formal clinical guidelines, and routine diabetes screening for all patients with fungal foot infections is not currently supported by our data. Instead of universal screening, clinicians should maintain a high index of suspicion and consider opportunistic HbA1c testing primarily in patients who present with other metabolic risk factors or infections that are particularly recalcitrant to standard treatment. Further evidence from prospective studies is required to define the exact criteria for targeted screening in this population.

## Data Availability

The data used in this study are based on pseudonymised individual-level health records from Danish national registers. Due to Danish data protection regulations, the datasets are not publicly available and cannot be shared by the authors. Access to the data requires project-specific approval from the Danish Health Data Authority and a valid data processing agreement
