# Supplementary material for: Can Foot Dermatophyte Infections Signal Future Diabetes Risk? Findings from a Register-based Study
Source: Acta Derm Venereol. 2026 Mar 9;106:0125. doi: 10.2340/actadv.v106.adv-2025-0125 (PMC12969967; doi:10.2340/actadv.v106.adv-2025-0125)
Supplement: Supplementary file 1. [file ActaDv-106-0125-s0001.docx]

# SUPPLEMENTAL TABLE LEGENDS

## **Table SI. Risk of type 2 diabetes in relation to number of positive dermatophyte PCR tests**

Among individuals in the exposed group, we examined whether undergoing three or more dermatophyte PCR tests was associated with an increased risk of developing diabetes.

| Variable | IRR with 95% CI | p-value |
| --- | --- | --- |
| ≥ 3 positive PCR tests versus ≤2 positive PCR tests | 1.01 (0.71 – 1.43) | 0.97 |

*Incidence rate ratio from Poisson regression.*

## **Table SII. Association between dermatophyte infections and development of prediabetes**

In the crude analysis, no significant association was found between exposure and risk of prediabetes (IRR 0.99, 95% CI: 0.94–1.05, p = 0.838).
Adjusting for sex did not change the result (IRR 0.97, 95% CI: 0.90–1.04, p = 0.334).

| Variable | IRR with 95% CI | p-value |
| --- | --- | --- |
| Crude  Adjusted for sex | 1.00 (0.94 – 1.05)  0.97 (0.90 – 1.04) | 0.87  0.34 |
